# Parental adherence to healthy lifestyles in relation to the risk of obesity in offspring: A prospective cohort study in China

**DOI:** 10.7189/jogh.13.04181

**Published:** 2023-12-20

**Authors:** Ye Qi, Rongxia Lv, Mengjia Zhao, Yuhui Huang, Yaodan Zhang, Hangrui Zhang, Zhihui Li, Peng Jia, Huanmei Zhang, Zhenyu Yang, Jianqiang Lai, Peige Song, Changzheng Yuan

**Affiliations:** 1 School of Public Health, the Second Affiliated Hospital, Zhejiang University School of Medicine, Hangzhou, Zhejiang, China; 2 Vanke School of Public Health, Tsinghua University, Beijing, China; 3 Department of Global Health and Population, Harvard T.H. Chan School of Public Health, Boston, Massachusetts, USA; 4 School of Resource and Environmental Sciences, Wuhan University, Wuhan, China; 5 International Institute of Spatial Lifecourse Health, Wuhan University, Wuhan, China; 6 National Institute for Nutrition and Health, Chinese Center for Disease Control and Prevention, Beijing, China; 7 School of Public Health and Women’s Hospital, Zhejiang University School of Medicine, Hangzhou, Zhejiang, China; 8 Department of Nutrition, Harvard T.H. Chan School of Public Health, Boston, Massachusetts, USA

## Abstract

**Background:**

While maternal adherence to a healthy lifestyle was shown to be associated with a lower risk of obesity in offspring, the potential role of overall parental lifestyles has not yet been explored. We aimed to address this gap by exploring whether parental adherence to an overall healthy lifestyle was associated with a lower risk of obesity in offspring.

**Methods:**

We included 5881 children and adolescents aged 6-15 years at enrolment in the 2010, 2012, and 2014 waves of the China Family Panel Studies (CFPS) who were free of obesity and followed them until 2020. Parental healthy lifestyle score at study baseline was composed of five modifiable lifestyle factors (0-5; 1 for each): never smoking, non-habitual drinking, weekly exercise, modified dietary diversity score ≥5 points, and body mass index (BMI) of 18.5-23.9 kg/m^2^. We defined obesity according to the age- and gender-specific cutoffs by the BMI percentile curves for Chinese children aged 6-18 years. We used multivariable Cox proportional hazard models to examine the association between parental healthy lifestyle score (both as continuous and categorical variables) and risk of offspring obesity.

**Results:**

Overall, 597 (10.2%) offspring developed obesity during a median follow-up of 6 years. Compared to the lowest tertile of parental healthy lifestyle score, participants in the highest tertile had a 42% (hazard ratio (HR) = 0.58; 95% confidence interval (CI) = 0.45-0.74) lower risk of obesity. Both maternal (HR = 0.75; 95% CI = 0.61-0.92) and paternal (HR = 0.73; 95% CI = 0.60-0.89) healthy lifestyle scores were associated with lower risks of obesity in offspring. For specific lifestyle factors, we observed beneficial associations for paternal diverse diet (HR = 0.73; 95% CI = 0.60-0.88) and healthy BMI (HR = 0.65; 95% CI = 0.55-0.78).

**Conclusions:**

Adherence to an overall parental healthier lifestyle was associated with a lower risk of obesity in childhood and adolescence. This finding highlights the potential benefits of promoting a healthy lifestyle among parents for the primary prevention of offspring obesity.

Obesity in childhood and adolescence has become a major public health issue worldwide [1-4], especially in China, where the prevalence of obesity has increased rapidly in the past few decades, rising to 7.9% in children and adolescents aged 6-17 years in 2015-2019 [5]. Childhood obesity, specifically, was found to be associated with higher risks of developing cardiometabolic disorders in adulthood, including obesity, cardiovascular disease, and premature death [6-13]. Therefore, it is crucial to identify the modifiable risk factors for the prevention of obesity in childhood.

Previous studies have demonstrated that the development of obesity in children and adolescents is highly related to unhealthy lifestyle behaviors [14,15], including unhealthy dietary habits, insufficient physical activity, and prolonged sedentary behavior [16]. Parents’ lifestyle behaviors have also been linked with risk of developing obesity in offspring, where maternal unhealthy diet, less physical activity, smoking, and drinking were found to be with a higher risk of obesity in offspring during early childhood [17-30]. Potential mechanisms involve offspring imitating parental lifestyle behaviors (eg, dietary habits or physical activity behavior) [31,32], and potential effects of parental lifestyles through epigenetic inheritance [33]. Nevertheless, research on the role of overall parental lifestyles, including the potential synergetic effect of different lifestyles, is very limited. Two previous cohort studies of mother-child pairs in the United States have observed a positive association between maternal overall healthy lifestyles (including diet, body mass index, exercise, smoking, and drinking) and a lower risk of offspring obesity from early childhood to young adulthood [18]. Although studies regarding paternal lifestyles are limited, paternal unhealthy diet and smoking were also found to be with offspring obesity [17,29]. Nevertheless, the potential role of overall parental healthy lifestyles in offspring obesity remains to be determined.

To address this gap, we aimed to investigate whether parental adherence to an overall healthy lifestyle was associated with a lower risk of offspring obesity during childhood and adolescence, based on data from parent-child pairs enrolled in the China Family Panel Studies (CFPS).

## METHODS

### Study population

We retrieved data for this prospective cohort study from the China Family Panel Studies (CFPS), a nationwide, comprehensive, and longitudinal social survey launched by Peking University, covering 25 provinces and representing 95% of the Chinese population. The first survey was conducted in 2010 and followed up every two years. It collected information about economic activity, education and work status, family relationships, population migration, physical and mental health, and other characteristics at community, household, and individual levels via face-to-face interviews [34]. The participants were dynamically updated with the birth and accession of new members and the death of old members. More details have been described elsewhere [34], while the data are available online [35]. The CFPS was approved by the Biomedical Ethics Review Committee of Peking University (IRB00001052-14010).

**Figure 1:**
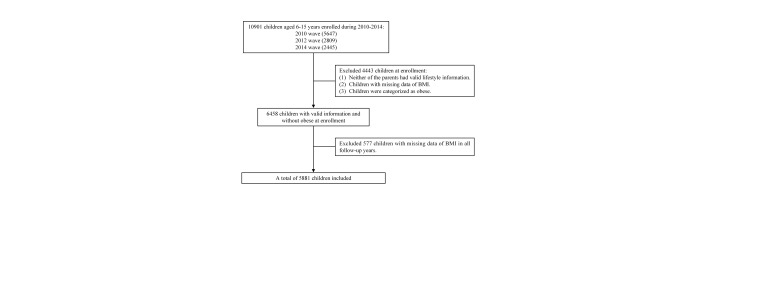
Flowchart of the study population.

We included 10 901 children and adolescents aged 6-15 years in 2010, 2012, and 2014 waves with available information on parental lifestyles, among whom 6458 had valid data on parental lifestyle information and were free of obesity at enrolment. We excluded a further 577 participants without body mass index (BMI) measures in any follow-up years (until 2020), resulting in a final sample of 5881 participants (**Figure 1**).

### Assessment of lifestyle factors in parents

All lifestyle factors of parents were collected through the questionnaire. Participants were asked how often, on average, they had consumed eight food items (such as meat and fish) over the past month in the 2010 wave, except for the 2012 and 2014 waves, where they were only asked whether they had consumed these eight food groups over the past week. Based on the Dietary Guidelines for Chinese Residents and the Dietary Diversity Score [36,37], parents received 1 point for each of the following six food groups they had weekly consumed and 0 points otherwise: fish and other aquatic products, fresh vegetables and fruits, dairy products, bean products, and eggs. We defined the modified Dietary Diversity Score by the sum of the scores, ranging from 0 (least healthy) to 6 (most healthy).

Participants were queried about their smoking history (including current and previous smoking status) and alcohol consumption (obtained by asking parents whether they had consumed alcoholic beverages more than three times a week in the last month, defined as habitual drinking). Physical activity was assessed by the average frequency and duration per week during the 2010 wave and only exercise frequency within the last non-vacation month in the 2012 and 2014 waves. BMI was calculated by dividing their weight (kg) by height squared (m^2^).

### Definition of the healthy lifestyle score

Based on previous reports [16,18,23], we considered the following five low-risk lifestyle factors: modified dietary diversity score equal to or greater than 5 points (about the top 40%), never smoking, non-habitual drinking (no more than three times per week in the last month), exercising every week, and having a normal BMI of 18.5-23.9 kg/m^2^. For each of the five low-risk factor, parents received a score of 1 and 0 otherwise, the sum of which constructed the healthy lifestyle score (range = 0-5), with a higher score indicating a healthier lifestyle. We calculated parental healthy lifestyle scores as the average of the maternal and paternal healthy lifestyle scores. If only one parent had completed the corresponding questionnaire, we defined the parental healthy lifestyle scores as per the available maternal/paternal one.

### Assessment of outcome

BMI in offspring was calculated by use of their self-reported weight and height, for children of 10 years old and over, or by reports from their caregivers for children under the age of 10 years. To define obesity in childhood and adolescence, we used the age- and sex-specific cutoffs by the BMI percentile curves for Chinese children aged 6-18 years developed by the National Health Commission of the People’s Republic of China (Screening for overweight and obesity among school-age children and adolescents (WS/T 586-2017) and Screening standard for malnutrition of school-age children and adolescents (WS/T 456-2014)). For adult offspring, we categorised BMI into the following four groups based on Chinese guidelines [38]: underweight (<18.5 kg/m^2^), normal weight (18.5-23.9 kg/m^2^), overweight (24.0-27.9 kg/m^2^), and obesity (≥28.0 kg/m^2^).

### Assessment of covariates

Offspring factors included age (continuous), gender (boys or girls), and living with parents (no, living with mother only, father only, or both parents), alongside self-reported lifestyle information such as exercise, diet, and sleep (with the first two having questions and low-risk definitions similar to those of parents). For sleep duration, we regarded 9-11 hours per night for offspring aged 6-13 years and 8-10 hours per night for those aged 14-15 years as low-risk factors. The combination of the above three lifestyle factors constructed a healthy lifestyle score for offspring (range = 0-3). Parental characteristics included age (binary), nationality (Han or ethnic minorities), education levels (illiterate/semi-literate, primary school, junior high school, or high school and above), employment status (employed or unemployed), and chronic disease history (yes or no). Chronic diseases included cardiovascular disease, hypertension, cerebrovascular disease, etc. Household characteristics included residency (urban or rural) and annual household income per capita on a log scale (binary).

### Statistical analysis

We examined the normality of data distribution using the Skewness-Kurtosis test, presenting continuous variables as means with standard deviations (SDs) or medians with interquartile ranges (IQRs) accordingly, and categorical variables as frequencies with percentages. We compared the baseline characteristic differences between participants grouped by tertiles of healthy lifestyle scores using analysis of variance (continuous variables with normal distribution), Kruskal-Wallis (continuous variables with non-normal distribution), or χ^2^ tests (categorical variables). We assigned missing categorical covariates to a separate group and gave median values to continuous variables of covariates. The follow-up period was drawn from the baseline (2010, 2012, and 2014 waves) until one of the following events, whichever came first: onset of obesity in the offspring, loss to follow-up, or being censored at the end of the study (2020 wave). We set up multivariable Cox proportional hazard models to explore the association between parental healthy lifestyle scores and the risk of obesity in offspring, regarding the former as both continuous (per 1-unit increments) and categorical variables. We adjusted for offspring’s age and gender in model 1, and additionally for offspring’s living with mother or father status; maternal age, nationality, education level, employment status, and chronic disease history; paternal age, nationality, education level, employment status, and chronic disease history; residency and household income per capita on a log scale in model 2. We also conducted linear trend tests in the corresponding models. We then evaluated the relationships between the overall or individual maternal or paternal healthy lifestyles with the risk of obesity in offspring. Since maternal and paternal healthy lifestyle scores were too concentrated in a certain score to be classified into three categories, we divided them into two groups according to the median as the cutoff value.

In secondary analyses, we evaluated the effect modification by subgroups stratified by offspring’s age (6-10 years or 11-15 years), gender (boys or girls), residency (urban or rural), household income per capita (low or high), parental education level (primary school and below or junior high school and above), and parental employment status (employed or unemployed). We also performed several sensitivity analyses to identify the robustness of associations. Based on model 2, we used the modified healthy lifestyle score (excluding BMI) ranging from 0 to 4; additionally adjusted for offspring’s lifestyle score; adjusted for cluster effects of households using the mixed linear model; only included one child from the same family; excluded parents with chronic disease history; excluded children who were underweight at baseline; defined study outcome as overweight and obesity, and; included children whose parents both have completed lifestyle information.

We considered a two-sided *P*-value <0.05 as statistically significant. All analyses were conducted using Stata, version 14.1 (StataCorp LLC, College Station, Texas, USA) and R, version 4.1.3 (R Core Team, Vienna, Austria).

## RESULTS

### Baseline characteristics

We included 5881 participants with a mean age of 10.2  years (SD = 3.0), of whom 51.9% were boys and 88.5% were Han. The average maternal and paternal ages were 36.8 (SD = 5.3), and 38.7 (SD = 5.7) years, respectively. The median overall parental healthy lifestyle score was 2.5, with 2, 3, and 4 scores for the lowest, medium, and highest tertiles, respectively. In general, offspring with higher parental healthy lifestyle scores were more likely to be younger and Han, while parents with higher healthy lifestyle scores had higher education levels and household income, and were more likely to live in urban areas (**Table 1** and Table S1 in the **Online Supplementary Document**).

**Table 1:** Baseline characteristics of study participants*

		Tertiles of the healthy lifestyle score†			
**Characteristics**	**Overall**	**T1**	**T2**	**T3**	***P*-value**‡
**Parental healthy lifestyle score, median**	2.5	2	3	4	
**Offspring characteristics**					
Number of offspring	5881	3060	1585	1236	
Age in years, meaan (SD)	10.2 (3.0)	10.3 (3.0)	10.2 (3.0)	9.9 (3.0)	0.001
BMI, kg/m^2^, mean (SD)	16.2 (3.1)	16.3 (3.2)	16.1 (3.0)	15.9 (2.9)	0.001
Gender – boys	3050 (51.9)	1568 (51.2)	834 (52.6)	648 (52.4)	0.61
Nationality – Han	3398 (88.5)	1675 (85.2)	945 (91.6)	778 (92.2)	<0.001
Living with parents					<0.001
*No*	1232 (21.0)	660 (21.6)	290 (18.3)	282 (22.9)	
*Living with mother only*	170 (2.9)	72 (2.4)	69 (4.4)	29 (2.3)	
*Living with father only*	586 (10.0)	291 (9.5)	185 (11.7)	110 (8.9)	
*Both parents*	3877 (66.1)	2026 (66.5)	1039 (65.6)	812 (65.9)	
**Maternal characteristics**					
Age in years, mean (SD)	36.8 (5.3)	36.9 (5.5)	36.7 (5.4)	36.6 (5.1)	0.03
BMI, kg/m^2^					<0.001
*<18.5*	476 (9.0)	355 (13.4)	85 (5.8)	36 (3.1)	
*18.5-23.9*	3421 (64.7)	1323 (49.8)	1094 (75.3)	1004 (85.3)	
*24-27.9*	1151 (21.8)	807 (30.4)	220 (15.1)	124 (10.5)	
*≥28*	240 (4.5)	173 (6.5)	54 (3.7)	13 (1.1)	
Nationality – Han	4695 (88.9)	2267 (85.4)	1327 (91.5)	1101 (93.6)	<0.001
Education					<0.001
*Illiterate/Semi-literate*	1455 (27.5)	923 (34.7)	370 (25.5)	162 (13.8)	
*Primary school*	1409 (26.6)	745 (28.0)	415 (28.6)	249 (21.2)	
*Junior high school*	1607 (30.4)	743 (28.0)	460 (31.7)	404 (34.3)	
*High school and above*	817 (15.5)	247 (9.3)	208 (14.3)	362 (30.8)	
Chronic disease history – Yes	597 (11.3)	303 (11.4)	186 (12.8)	108 (9.2)	0.01
Employment status – employed	3279 (63.0)	1632 (62.3)	893 (62.7)	754 (64.9)	0.31
Lifestyle factors					
*Never smoking*	5036 (95.2)	2461 (92.6)	1411 (97.1)	1164 (98.9)	<0.001
*No habitual drinking*	5163 (97.6)	2558 (96.2)	1438 (99.0)	1167 (99.2)	<0.001
*Weekly exercise*	911 (17.2)	177 (6.7)	235 (16.2)	499 (42.4)	<0.001
*Diverse diet*	2042 (38.6)	456 (17.2)	603 (41.5)	983 (83.5)	<0.001
*Healthy BMI*	3421 (64.7)	1323 (49.8)	1094 (75.3)	1004 (85.3)	<0.001
**Paternal characteristics**					
Age in years, mean (SD)	38.7 (5.7)	38.8 (5.9)	38.6 (5.6)	38.3 (5.3)	0.07
BMI, kg/m^2^					<0.001
*<18.5*	198 (4.1)	168 (6.0)	24 (2.2)	6 (0.6)	
*18.5-23.9*	3002 (62.2)	1510 (54.0)	787 (71.8)	705 (75.4)	
*24-27.9*	1288 (26.7)	887 (31.7)	229 (20.9)	172 (18.4)	
*≥28*	340 (7.0)	232 (8.3)	56 (5.1)	52 (5.6)	
Nationality – Han	4302 (89.3)	2410 (86.4)	1033 (94.3)	859 (92.0)	<0.001
Education					<0.001
*Illiterate/Semi-literate*	835 (17.3)	654 (23.4)	101 (9.2)	80 (8.6)	
*Primary school*	1265 (26.2)	837 (29.9)	263 (24.0)	165 (17.6)	
*Junior high school*	1758 (36.4)	947 (33.9)	482 (44.0)	329 (35.2)	
*High school and above*	970 (20.1)	359 (12.8)	250 (22.8)	361 (38.6)	
Chronic disease history – Yes	438 (9.1)	264 (9.4)	95 (8.7)	79 (8.4)	0.57
Employment status – employed	3698 (77.5)	2081 (75.5)	870 (80.0)	747 (80.6)	<0.001
Lifestyle factors					<0.001
*Never smoking*	1196 (24.8)	366 (13.1)	385 (35.1)	445 (47.6)	<0.001
*No habitual drinking*	3269 (67.7)	1635 (58.5)	834 (76.1)	800 (85.6)	<0.001
*Weekly exercise*	954 (19.8)	230 (8.2)	255 (23.3)	469 (50.2)	<0.001
*Diverse diet*	2124 (44.0)	729 (26.1)	605 (55.2)	790 (84.5)	<0.001
*Healthy BMI*	3002 (62.2)	1510 (54.0)	787 (71.8)	705 (75.4)	<0.001
**Family characteristics**					<0.001
Residency – rural	3585 (61.2)	2143 (70.4)	937 (59.3)	505 (41.0)	<0.001
Household income per capita in 2010, log scale, mean (SD)	8.5 (1.1)	8.3 (1.1)	8.5 (1.1)	8.9 (1.1)	<0.001

SD – standard deviation, IQR – interquartile range, BMI – body mass index, T – tertile

*Data presented as n (%) unless specified otherwise.

†T1: lowest tertile of the healthy lifestyle score. T2: medium tertile of the healthy lifestyle score. T3: highest tertile of the healthy lifestyle score.

‡The differences between tertiles of healthy lifestyle score were compared by Kruskal-Wallis (continuous variables) and χ^2^ test (categorical variables).

### Parental lifestyle and risk of obesity in offspring

We identified 597 (10.2%) offspring who developed obesity during a median of six years of follow-up. In the multivariable-adjusted model (model 2), compared to the lowest tertile, offspring in the highest tertile of parental healthy lifestyle scores had a 42% lower risk of obesity (hazard ratio (HR) = 0.58; 95% confidence interval (CI) = 0.45-0.74, *P*-value for <0.001) (**Table 2**). Moreover, each one-unit increment in the parental healthy lifestyle score was associated with a 23% lower risk of offspring obesity (HR = 0.77; 95% CI = 0.69-0.86).

**Table 2:** Association of parental healthy lifestyle score with risk of obesity in offspring

	Tertiles of healthy lifestyle score*				
	**T1**	**T2**	**T3**	**Healthy lifestyle score (continuous variable)**	***P*-value for trend**†
**Parental healthy lifestyle score, median**	2	3	4	2.5	
**Number of offspring**	3060	1585	1156	5881	
**Cases**	370	147	80	597	
**Person-years**	19 704	10 366	7896	37966	
**Cases/PYs**	18.78	18.18	10.13	15.72	
**Model 1, HR (95% CI)**‡	ref	0.74 (0.61-0.90)	0.49 (0.38-0.62)	0.71 (0.65-0.79)	<0.001
**Model 2, HR (95% CI)**‡	ref	0.87 (0.71-1.06)	0.58 (0.45-0.74)	0.77 (0.69-0.86)	<0.001

HR – hazard ratio, CI – confidence interval, cases/PYs – incidence rate per 1000 person-years, T – tertile, ref – reference

*T1: lowest tertile of the healthy lifestyle score. T2: medium tertile of the healthy lifestyle score. T3: highest tertile of the healthy lifestyle score.

†Calculated by entering stratum-specific median values for all participants in each tertile of healthy lifestyle score as continuous variables in Multivariable Cox proportional hazard models.

‡Model 1 adjusted for offspring’s age, gender. Model 2 adjusted for offspring’s age, gender, living with parents; maternal age, nationality, education level, employment status, chronic disease history; paternal age, nationality, education level, employment status, chronic disease history; residency, household income per capita.

**Figure 2:**
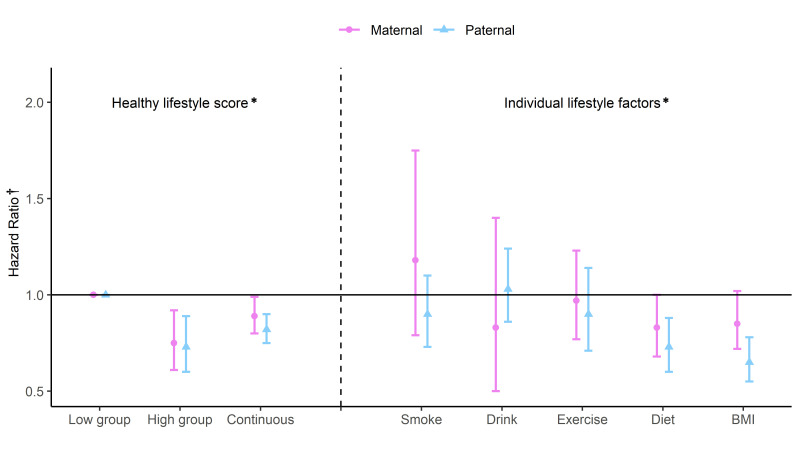
Association between maternal and paternal healthy lifestyle and risk of obesity in offspring. *Low group: less than or equal to median healthy lifestyle score (maternal score ≤3; paternal score ≤2). High group: higher than median healthy lifestyle score (maternal score >3; paternal score >2). Continuous: healthy lifestyle score per 1-unit increments. Smoke: never smoking. Drink: non-habitual drinking (no more than three times per week in the last month). Exercise: exercise weekly. Diet: modified dietary diversity score greater than or equal to 5 points (about the top 40%). BMI: a healthy body mass index of 18.5-23.9. †Model adjusted for offspring’s age, gender, living with mother/father; maternal/paternal age, nationality, education level, employment status, chronic disease history; residency, household income per capita. For individual lifestyle factors, model additionally adjusted for other lifestyle factors. BMI – body mass index.

We observed a similar association in terms of maternal or paternal lifestyles on offspring obesity (**Figure 2** and Table S2 in the **Online Supplementary Document**). Compared to the lowest group, the highest groups of maternal (HR = 0.75; 95% CI = 0.61-0.92) and paternal (HR = 0.73; 95% CI = 0.60-0.89) healthy lifestyle scores were associated with lower risks of obesity in offspring, after adjusting for offspring, maternal, paternal and household covariates. These results were attenuated but still significant when the healthy lifestyle score was assigned as a continuous variable, with an 11% lower risk of offspring obesity for a one-unit increment in maternal healthy lifestyle score (HR = 0.89; 95% CI = 0.80-0.99) and an 18% lower risk of offspring obesity for each one unit higher in paternal healthy lifestyle score (HR = 0.82; 95% CI = 0.75-0.90). In particular, children of fathers who adhered to diverse diets (HR = 0.73; 95% CI = 0.60-0.88) and had healthy BMI (HR = 0.65; 95% CI = 0.55-0.78) had 27% lower risk or 35% lower risk of offspring obesity, respectively. Furthermore, maternal adherence to a diverse diet (HR = 0.83; 95% CI = 0.68-1.00) and having a healthy BMI (HR = 0.85; 95% CI = 0.72-1.02) also demonstrated a negative association with offspring obesity (**Figure 2** and Table S3 in the **Online Supplementary Document**).

### Subgroup and sensitivity analyses

**Figure 3:**
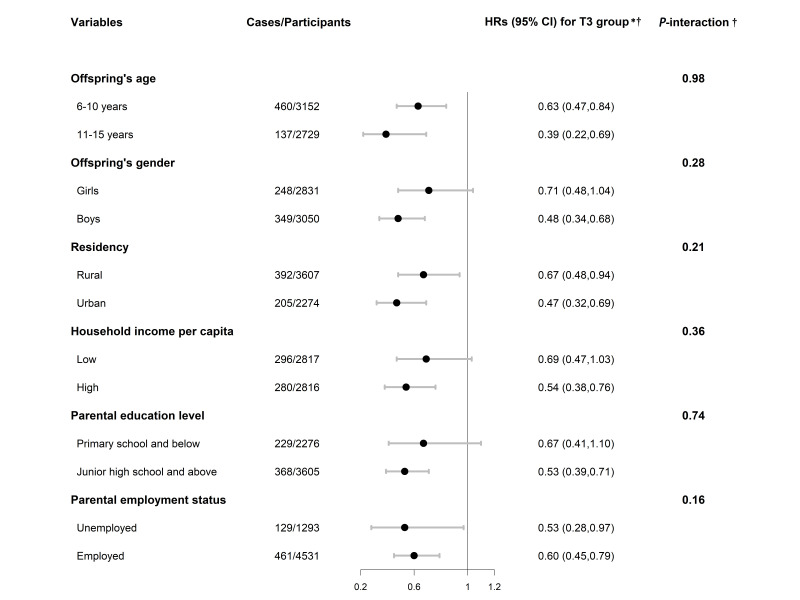
Subgroup analysis of parental healthy lifestyle score with risk of obesity in offspring. *T3: highest tertile of the healthy lifestyle score (reference: lowest tertile of the healthy lifestyle score). †Model adjusted for offspring’s age, gender, living with parents; maternal age, nationality, education level, employment status, chronic disease history; paternal age, nationality, education level, employment status, chronic disease history; residency, household income per capita. HR – hazard ratio, CI – confidence interval.

We detected no significant effect modifications by major subgroups, including offspring’s age, gender, residency, household income, parental education, and parental employment (*P*-values for interaction >0.05) (**Figure 3** and Table S4 in the **Online Supplementary Document**). The results remained robust in a series of sensitivity analyses after modifying healthy lifestyle scores (excluding BMI, with a range of 0-4), additionally adjusting offspring lifestyle scores, using the mixed linear model to additionally adjust for cluster effects of households, and excluding duplicated observations within families, parents with chronic disease history, and underweight offspring at baseline, as well as defining the study outcome as overweight and obesity, and including children whose parents both have completed lifestyle information (Table S5 in the **Online Supplementary Document**).

## DISCUSSION

In this nationwide prospective cohort study with a median follow-up of six years, parental adherence to an overall healthier lifestyle was associated with a lower risk of offspring obesity in childhood and adolescence, after adjustment for multiple offspring, maternal, paternal, and household factors. Both maternal and paternal healthy lifestyle scores were associated with lower risks of obesity in offspring, with significant contribution observed for paternal diverse diet and healthy BMI.

Previous studies have shown that lifestyles may play an important role in the development of obesity, with increasing attention being paid to the role of parental lifestyle in offspring obesity. Two prospective studies from the Nurses’ Health Study II (NHSII) and Growing Up Today Study (GUTS) quantified the role of combined maternal healthy lifestyles (characterised by a healthy BMI, high-quality diet, regular exercise, no smoking, and low-to-moderate alcohol intake) on obesity in offspring from early childhood to young adulthood [18]. Among 24 289 participants (aged 9-14 years at baseline), a 75% lower risk of obesity was observed comparing individuals with overall healthy to those with unhealthy maternal lifestyles. However, little is known about the association of overall parental healthy lifestyle with risk of offspring obesity. Here we examined the association between overall parental lifestyle factors and offspring obesity, emphasising the combined role of both parents, and observed that parental adherence to a healthy lifestyle (characterised by a diverse diet, no smoking, no habitual drinking, regular exercise, and normal BMI) was associated with a 42% lower risk of obesity in offspring. The results remained similar after adjustment for social and economic factors and offspring lifestyles. In line with the previous study [18], we observed a protective association between maternal adherence to a healthy lifestyle and the risk of obesity in offspring. We also found that paternal adherence to a healthy lifestyle was also inversely associated with risk of offspring obesity.

Several studies have been conducted to evaluate the roles of parental lifestyle factors on obesity in offspring, demonstrating that parental obesity was associated with a higher risk of obesity in offspring [15,39]. Conversely, few studies examined the role of parental diet in offspring obesity, and were primarily conducted before or during pregnancy; they suggested that adherence to a better maternal diet was associated with a reduced risk of obesity in childhood [40-42]. We found that normal BMI and diverse dietary intake in parents, especially fathers, were associated with a lower risk of offspring obesity. Moreover, studies have demonstrated that parental smoking before or during pregnancy could increase the risk of obesity in offspring [27-29,43]. Few studies have examined the relationship between parental exercise and drinking and offspring obesity. We did not find a significant association between maternal or paternal smoking, exercise, and drinking and obesity in offspring.

Although the mechanisms underlying the protective roles of parental healthy lifestyles on offspring obesity remain unclear, parental lifestyle behaviours may critically impact their children’s lifestyle and subsequently modulate their development of obesity. Additionally, parental diet may influence children’s dietary habits [44,45], thereby changing their nutritional status and ultimately leading to obesity [15,46]. A poor paternal diet was found to be linked with an increased risk of obesity possibly through epigenetic effects [33,47]. Further possible explanations for the influence of parental obesity on offspring obesity include regulation of epigenetic effects [48] and changes in obesity-related hormone levels, such as higher adiposity in parents and lower anorexigenic hormone in offspring [49,50]. Animal experiments have found that maternal obesity could promote morphology alteration of adipocytes in the foetus related to the development of offspring obesity [51]. In terms of physical activity, parents can influence their children’s physical activity through role modeling (being active themselves), material support (financial, logistic, co-participation), and encouragement, further influencing their children’s obesity [31,52].

This was a longitudinal study in which we followed a population-based cohort for up to ten years. We adjusted for a set of parental and family confounding factors and conducted a series of sensitivity analyses, enhancing the robustness of our results, while including detailed information about parental and offspring lifestyle factors, allowing us to conduct comprehensive analyses. However, we had limited data for some lifestyle factors; for example, we lacked comprehensive information on diet, while definitions of physical activity and alcohol consumption were relatively simple. Second, information on lifestyles and other covariates were self-reported, which may lead to inaccurate responses and recall bias. Third, as this is an observational study, we cannot exclude the possibility of uncontrolled and residual confounding effects. However, we performed different sensitivity analyses to test the robustness of our results. Lastly, the results from this study were observed among the Chinese children and adolescent population and may not be generalisable to other populations.

## CONCLUSIONS

We found that parental adherence to a healthier lifestyle was associated with a lower risk of offspring obesity in childhood and adolescence, wherein both paternal and maternal healthy lifestyles played beneficial roles in the relationship. These findings highlight the potential benefits of promoting a healthy lifestyle among parents for the prevention of offspring obesity and provide support for family- or parent-based intervention strategies for childhood obesity.

## Additional material


Online Supplementary Document
