# Supplementary material for: Parental adherence to healthy lifestyles in relation to the risk of obesity in offspring: A prospective cohort study in China
Source: J Glob Health. 2023 Dec 20;13:04181. doi: 10.7189/jogh.13.04181 (PMC10731135; doi:10.7189/jogh.13.04181)
Supplement: Online Supplementary Document [file jogh-13-04181-s001.pdf]

Online Supplementary Document

Table S1 Baseline characteristics of study participants [median (IQR)].

Table S2 Association between combined maternal or paternal healthy lifestyle score and risk of obesity in offspring.

Table S3 Association between individual maternal or paternal lifestyle factors with risk of obesity in offspring.

Table S4 Subgroup analysis of parental healthy lifestyle score with risk of obesity in offspring.

Table S5 Sensitivity analyses for the association of parental healthy lifestyle score with risk of obesity in offspring.

Table S1. Baseline characteristics of study participants [median (IQR)]

Characteristics	Overall	Tertiles of the healthy lifestyle score*			
		T1	T2	T3	***P* value†**
Offspring' age, years	10.0 (7.0, 13.0)	10.0 (8.0, 13.0)	10.0 (7.0, 13.0)	10.0 (7.0, 13.0)	0.001
Offspring' BMI, kg/m^2	16.2 (14.3, 17.8)	16.4 (14.5, 18.0)	16.0 (14.2, 17.8)	15.9 (14.0, 17.8)	0.001
Maternal age, years	37.0 (33.0,40.0)	37.0 (33.0, 40.0)	37.0 (33.0, 40.0)	36.0 (33.0, 40.0)	0.03
Paternal age, years	38.0 (35.0, 42.0)	38.0 (35.0, 42.0)	38.0 (35.0, 42.0)	38.0 (34.0, 42.0)	0.07
Household income per capita in 2010, log scale	8.6 (8.0, 9.2)	8.4 (7.8, 9.0)	8.6 (8.1, 9.2)	9.0 (8.4, 9.6)	<0.001

IQR – interquartile range, BMI – body mass index.

*T1: lowest tertile of the healthy lifestyle score. T2: medium tertile of the healthy lifestyle score. T3: highest tertile of the healthy lifestyle score.

†The differences between tertiles of healthy lifestyle score were compared by Kruskal-Wallis test.

Table S2. Association between combined maternal or paternal healthy lifestyle score and risk of obesity in offspring

	Healthy lifestyle score*		Healthy lifestyle score (continuous variable)	*P*-trend†
	Low	High		
	HR (95% CI)	HR (95% CI)	HR (95% CI)	
**Maternal**				
**Maternal healthy lifestyle score, median**	3	4	3	
**No. of offspring**	3622	1666	5288	
**Cases**	404	133	537	
**Person-years**	23830	10638	34468	
**Cases / PYs**	16.95	12.50	15.58	
Model 1‡	Ref.	0.68 (0.56,0.82)	0.83 (0.75,0.92)	<0.001
Model 2‡	Ref.	0.75 (0.61,0.92)	0.89 (0.80,0.99)	0.01
**Paternal**				
**Paternal healthy lifestyle score, median**	2	3	2	
**No. of offspring**	3119	1709	4828	
**Cases**	378	145	523	
**Person-years**	20102	11054	31156	
**Cases / PYs**	18.80	13.12	16.79	
Model 1‡	Ref.	0.67 (0.56,0.81)	0.79 (0.73,0.86)	<0.001
Model 2‡	Ref.	0.73 (0.60,0.89)	0.82 (0.75,0.90)	0.002

HR – hazard ratio, CI – confidence interval, Cases / PYs – incidence rate per 1000 person-years, Ref – reference.

*Low group: less than or equal to median healthy lifestyle score (maternal score ≤3; paternal score ≤2). High group: higher than median healthy lifestyle score (maternal score >3; paternal score >2). Continuous: healthy lifestyle score per 1-unit increments.

†*P*-trend: calculated by entering stratum-specific median values for all participants in each tertile of healthy lifestyle score as continuous variables in Multivariable Cox proportional hazard models.

‡Model 1 adjusted for offspring's age, gender. Model 2 adjusted for offspring's age, gender, living with mother/father; maternal/paternal age, nationality, education level, employment status, chronic disease history; residency, household income per capita.

Table S3. Association between individual maternal or paternal lifestyle factors with risk of obesity in offspring

Lifestyle factors*		No. of participants	Cases	Model 1†	Model 2†
				HR (95% CI)	HR (95% CI)
**Maternal**					
**Smoke**	0	252	26	Ref.	Ref.
	1	5036	511	1.17 (0.79,1.74)	1.18 (0.79,1.75)
**Drink**	0	125	15	Ref.	Ref.
	1	5163	522	0.86 (0.52,1.44)	0.83 (0.50,1.40)
**Exercise**	0	4377	451	Ref.	Ref.
	1	911	86	0.97 (0.77,1.23)	0.97 (0.77,1.23)
**Diet**	0	3246	358	Ref.	Ref.
	1	2042	179	0.83 (0.68,1.00)	0.83 (0.68,1.00)
**BMI**	0	1867	210	Ref.	Ref.
	1	3421	327	0.85 (0.72,1.01)	0.85 (0.72,1.02)
**Paternal**					
**Smoke**	0	3632	404	Ref.	Ref.
	1	1196	119	0.91 (0.74,1.11)	0.90 (0.73,1.10)
**Drink**	0	1559	174	Ref.	Ref.
	1	3269	349	1.04 (0.87,1.25)	1.03 (0.86,1.24)
**Exercise**	0	3874	433	Ref.	Ref.
	1	954	90	0.91 (0.72,1.14)	0.90 (0.71,1.14)
**Diet**	0	2704	330	Ref.	Ref.
	1	2124	193	0.74 (0.61,0.89)	0.73 (0.60,0.88)
**BMI**	0	1826	244	Ref.	Ref.
	1	3002	279	0.67 (0.56,0.79)	0.65 (0.55,0.78)

BMI – body mass index, HR – hazard ratio, CI – confidence interval, Ref – reference.

*"1" represents meeting the criteria for low-risk lifestyle factors. Smoke: never smoking. Drink: non-habitual drinking (no more than three times per week in the last month). Exercise: exercise weekly. Diet: modified dietary diversity score greater than or equal to 5 points (about the top 40%), while "0" is on the opposite. BMI: a healthy body mass index of 18.5-23.9.

†Model 1 adjusted for offspring's age, gender, living with mother/father; maternal/paternal age, nationality, education level, employment status, chronic disease history; residency, household income per capita. Model 2 additionally adjusted for other lifestyle factors.

Table S4. Subgroup analysis of parental healthy lifestyle score with risk of obesity in offspring

	No. of participants	Cases	Tertiles of healthy lifestyle score*			*P*-interaction†
			T1	T2	T3	
			HR (95% CI)	HR (95% CI)	HR (95% CI)	
**Offspring's age**						
6-10 years	3152	460	Ref.	0.91 (0.73,1.15)	0.63 (0.47,0.84)	0.98
11-15 years	2729	137	Ref.	0.67 (0.43,1.03)	0.39 (0.22,0.69)	
**Offspring's gender**						
Girls	2831	248	Ref.	0.85 (0.61,1.18)	0.71 (0.48,1.04)	0.28
Boys	3050	349	Ref.	0.86 (0.67,1.12)	0.48 (0.34,0.68)	
**Residency**						
Rural	3607	392	Ref.	0.96 (0.75,1.23)	0.67 (0.48,0.94)	0.21
Urban	2274	205	Ref.	0.71 (0.51,1.00)	0.47 (0.32,0.69)	
**Household income per capita**						
Low	2817	296	Ref.	0.99 (0.74,1.31)	0.69 (0.47,1.03)	0.36
High	2816	280	Ref.	0.85 (0.63,1.13)	0.54 (0.38,0.76)	
**Parental education level**						
Primary school and below	2276	229	Ref.	0.88 (0.62,1.26)	0.67 (0.41,1.10)	0.74
Junior high school and above	3605	368	Ref.	0.84 (0.66,1.07)	0.53 (0.39,0.71)	
**Parental employment status**						
Unemployed	1293	129	Ref.	1.17 (0.79,1.74)	0.53 (0.28,0.97)	0.16
Employed	4531	461	Ref.	0.80 (0.64,1.01)	0.60 (0.45,0.79)	

HR – hazard ratio, CI – confidence interval, Ref – reference.

*T1: lowest tertile of the healthy lifestyle score. T2: medium tertile of the healthy lifestyle score. T3: highest tertile of the healthy lifestyle score.

†Model adjusted for offspring's age, gender, living with parents; maternal age, nationality, education level, employment status, chronic disease history; paternal age, nationality, education level, employment status, chronic disease history; residency, household income per capita.

Table S5. Sensitivity analyses for the association of parental healthy lifestyle score with risk of obesity in offspring

	Tertiles of healthy lifestyle score*			Healthy lifestyle score (continuous variable)	***P*-trend†‡**
	**T1**	**T2**	**T3**		
	**HR (95% CI)**	**HR (95% CI)**	**HR (95% CI)**	**HR (95% CI)**	
**Sensitivity analysis 1**§	Ref.	0.87 (0.68,1.10)	0.72 (0.56,0.93)	0.84 (0.73,0.95)	0.01
**Sensitivity analysis 2**§	Ref.	0.68 (0.45,1.04)	0.39 (0.22,0.68)	0.55 (0.43,0.70)	0.001
**Sensitivity analysis 3**§	Ref.	0.81 (0.62.1.06)	0.46 (0.33,0.64)	0.69 (0.59,0.80)	<0.001
**Sensitivity analysis 4**§	Ref.	0.89 (0.71,1.13)	0.57 (0.43,0.77)	0.77 (0.68,0.88)	<0.001
**Sensitivity analysis 5**§	Ref.	0.88 (0.71,1.09)	0.60 (0.46,0.78)	0.76 (0.68,0.86)	<0.001
**Sensitivity analysis 6**§	Ref.	0.87 (0.69,1.09)	0.53 (0.40,0.72)	0.75 (0.65,0.85)	<0.001
**Sensitivity analysis 7**§	Ref.	0.88 (0.76,1.02)	0.70 (0.59,0.83)	0.84 (0.77,0.91)	<0.001
**Sensitivity analysis 8**§	Ref.	0.86 (0.68,1.08)	0.60 (0.45,0.79)	0.75 (0.65,0.86)	<0.001

HR – hazard ratio, CI – confidence interval, Ref – reference.

*T1: lowest tertile of the healthy lifestyle score. T2: medium tertile of the healthy lifestyle score. T3: highest tertile of the healthy lifestyle score.

†*P*-trend: calculated by entering stratum-specific median values for all participants in each tertile of healthy lifestyle score as continuous variables in Multivariable Cox proportional hazard models.

‡Model adjusted for offspring's age, gender, living with parents; maternal age, nationality, education level, employment status, chronic disease history; paternal age, nationality, education level, employment status, chronic disease history; residency, household income per capita.

§Sensitivity analysis 1: modifing healthy lifestyle score (excluding BMI). Sensitivity analysis 2: additionally adjusted for offspring's lifestyle score (N=3009). Sensitivity analysis 3: additionally adjusted for cluster effects of households using the Mixed linear model. Sensitivity analysis 4: excluding duplicated observations within families (N=4487). Sensitivity analysis 5: excluding parents with chronic disease history (N=4947). Sensitivity analysis 6: excluding children who were categorized as underweight at enrollment (N=4218). Sensitivity analysis 7: retreating the study outcome as overweight and obesity (N=5079). Sensitivity analysis 8: retained children whose parents both have complete lifestyle information (N=4235).
